# Functional diversification of teleost Fads2 fatty acyl desaturases occurs independently of the trophic level

**DOI:** 10.1038/s41598-019-47709-0

**Published:** 2019-08-01

**Authors:** Diego Garrido, Naoki Kabeya, Mónica B. Betancor, José A. Pérez, N. Guadalupe Acosta, Douglas R. Tocher, Covadonga Rodríguez, Óscar Monroig

**Affiliations:** 1 Departamento de Biología Animal, Edafología y Geología, Universidad de La Laguna, La Laguna, 38206 Santa Cruz de Tenerife Spain; 2 Department of Aquatic Bioscience, The University of Tokyo, Yayoi, Bunkyo-ku, Tokyo Japan; 3 Institute of Aquaculture, Faculty of Natural Sciences, University of Stirling, Stirling, FK9 4LA Scotland UK; 4 Instituto de Acuicultura Torre de la Sal, Consejo Superior de Investigaciones Científicas (IATS-CSIC), 12595 Ribera de Cabanes, Castellón, Spain

**Keywords:** Fatty acids, Ecology, Animal physiology

## Abstract

The long-chain (≥C_20_) polyunsaturated fatty acid biosynthesis capacity of fish varies among species, with trophic level hypothesised as a major factor. The biosynthesis capacity is largely dependent upon the presence of functionally diversified fatty acyl desaturase 2 (Fads2) enzymes, since many teleosts have lost the gene encoding a Δ5 desaturase (Fads1). The present study aimed to characterise Fads2 from four teleosts occupying different trophic levels, namely *Sarpa salpa*, *Chelon labrosus*, *Pegusa lascaris* and *Atherina presbyter*, which were selected based on available data on functions of Fads2 from closely related species. Therefore, we had insight into the variability of Fads2 within the same phylogenetic group. Our results showed that Fads2 from *S*. *salpa* and *C*. *labrosus* were both Δ6 desaturases with further Δ8 activity while *P*. *lascaris* and *A*. *presbyter* Fads2 showed Δ4 activity. Fads2 activities of herbivorous *S*. *salpa* are consistent with those reported for carnivorous Sparidae species. The results suggested that trophic level might not directly drive diversification of teleost Fads2 as initially hypothesised, and other factors such as the species’ phylogeny appeared to be more influential. In agreement, Fads2 activities from *P*. *lascaris* and *A*. *presbyter* were similar to their corresponding phylogenetic counterparts *Solea senegalensis* and *Chirostoma estor*.

## Introduction

The crucial roles of long-chain (≥C_20_) polyunsaturated fatty acids (LC-PUFA) such as eicosapentaenoic acid (EPA, 20:5n-3), docosahexaenoic acid (DHA, 22:6n-3) and arachidonic acid (ARA, 20:4n-6) in human nutrition and their potential health benefits have been extensively studied^1–4^. These lipid components are implicated in a range of structural, functional and signaling processes^1,4,5^. LC-PUFA, particularly those of the n-3 series, are naturally produced by lower trophic organisms of the marine food web including single-celled organisms^6,7^ and invertebrates^8^, and are accumulated in higher trophic organisms. Consequently, fish are the main sources of these beneficial LC-PUFA in the human food basket^9,10^. However, current growth of the human population, which will probably exceed 9 billion by 2050, along with overexploitation of traditional fisheries, make aquaculture activities a keystone in order to meet future dietary demand for LC-PUFA^11^. While farmed fish products have been good sources of LC-PUFA for human consumers when high fishmeal and fish oil levels have been included in aquafeed formulations, increasing levels of inclusion of non-marine ingredients in current aquafeed formulations can potentially lead to growth and health issues in species with low capacity to use alternative plant-based ingredients and greatly reduces the contents of healthy LC-PUFA for consumers^12^.

One approach to address this problem is bioengineering of terrestrial plants to produce LC-PUFA and, therefore, help maintain their level in aquafeeds and in turn conserve the quality of farmed fish flesh^13,14^. An alternative to bioengineering could be the use of marine macro and microalgae, although there are some drawbacks such as limited production and high cost, as well as the potential presence of anti-nutritional factors^12,15^. Alternatively, the culture of fish species with high capability to biosynthesise LC-PUFA from shorter chain (C_18_) polyunsaturated fatty acids (PUFA) (e.g. 18:3n-3 and 18:2n-6) abundant in terrestrial vegetable oils could also be a sensible approach to support the sustainable expansion of aquaculture. However, with a few exceptions like salmonids, most species currently farmed in North America and Europe are marine carnivores with no, or very limited, capacity for the endogenous production (biosynthesis) of LC-PUFA from their C_18_ PUFA precursors^15,16^. The trophic level of fish species, along with their habitat (freshwater vs. marine) and trophic ecology, have all been suggested to influence the capacity for biosynthesis of LC-PUFA of fish^16,17^ and, consequently, the way essential fatty acid (FA) requirements are satisfied by the diet. In species with high capacity to convert C_18_ PUFA to LC-PUFA, essential FA can be satisfied by including vegetable oils containing C_18_ PUFA in the diet. In contrast, species with low capacity to convert C_18_ PUFA to LC-PUFA require a dietary supply of the latter to satisfy their essential FA demands, with this typically achieved by including fish oil^16,18–21^.

**Figure 1:**
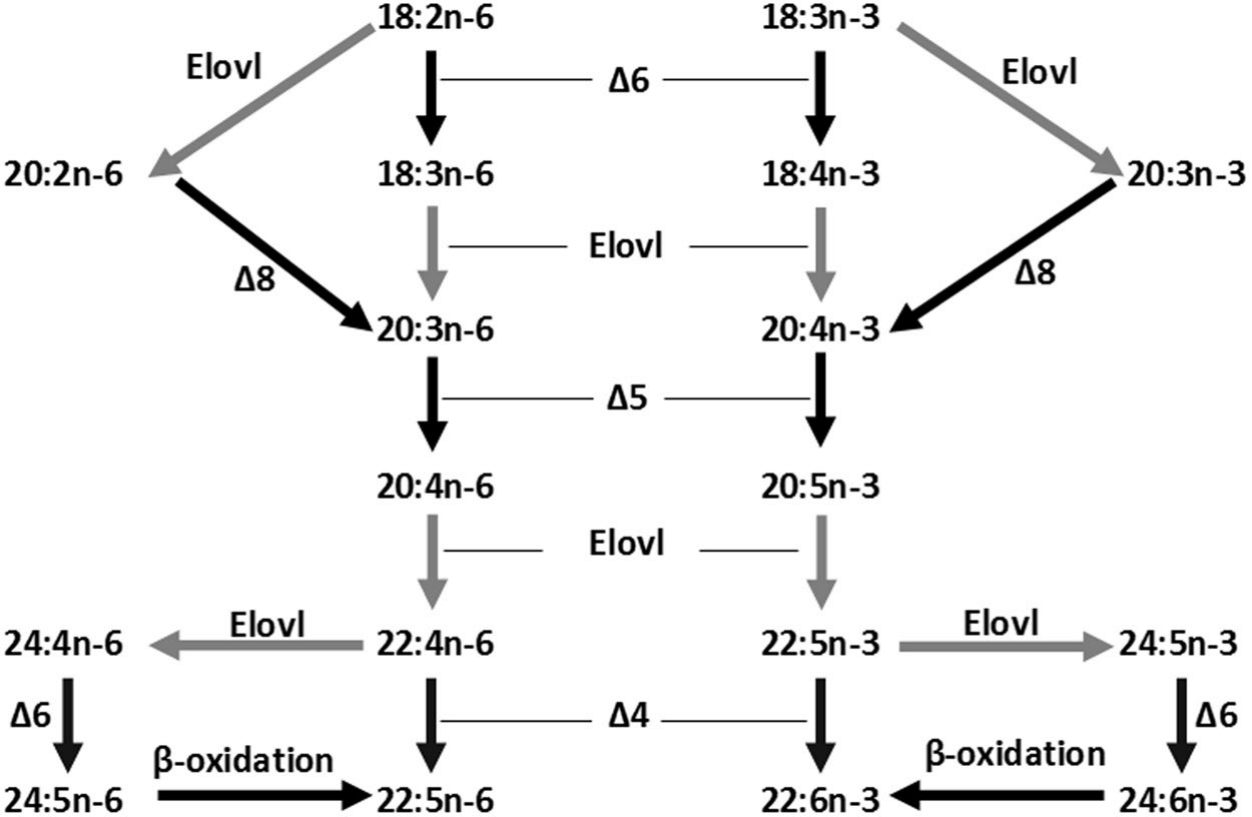
Biosynthetic pathways of LC-PUFA from the precursors linoleic acid (18:2n-6) and α-linolenic acid (18:3n-3) in teleosts. Black and grey arrows show reactions catalysed by fatty acyl desaturase (“Δx”) and fatty acyl elongase (“Elovl”) activities, respectively.

The biosynthesis of LC-PUFA from C_18_ PUFA precursors is carried out in vertebrates by aerobic desaturase and elongase enzymes^16^ (Fig. 1). Fatty acyl desaturases (Fads or Δx) introduce double bonds between a pre-existing double bond and the carboxylic group and are therefore termed “front-end” desaturases. Moreover, elongation of very long chain fatty acids (Elovl) proteins are responsible for the initial condensation reaction of the elongation pathway that results in the addition of two carbons to pre-existing FA substrate^16^. In mammals, two Fads, namely FADS1 and FADS2, are the main desaturase enzymes involved in LC-PUFA biosynthesis^16^. Basically, mammalian FADS1 possesses Δ5 desaturase activity responsible for the synthesis of EPA and ARA from 20:4n-3 and 20:3n-6, respectively. FADS2, while mostly possesses Δ6 desaturase activity that catalyzes the desaturation of C_18_ PUFA, namely 18:3n-3 and 18:2n-6 (Fig. 1), it has been also shown to catalyse Δ4 desaturation to yield DHA acid and 22:5n-6 acid in human cells^22^. Importantly, FADS2 also has an important role in mammals in the synthesis of DHA from EPA *via* the so-called “Sprecher pathway”, which comprises two sequential elongations from EPA to 24:5n-3, a Δ6 desaturation leading to 24:6n-3 and a final chain-shortening (partial β-oxidation) to produce DHA^23^.

In contrast to mammals, virtually all Fads isolated from Teleostei species are phylogenetically identified as *fads2* orthologues suggesting that they have lost the *fads1* gene during evolution^24^, with the sole exception of basal teleosts like the Japanese eel *Anguilla japonica*, which retained a *fads1* encoding a ∆5 desaturase^25^. Many teleost Fads2 show Δ6 desaturase activity along with Δ8 desaturase activity similar to mammalian FADS2^26,27^. However, in contrast to mammalian FADS2, a greater functional diversification has been described among teleost Fads2, thus enabling some species to compensate for loss of *fads1* and thus enhance their LC-PUFA biosynthetic capacities. Bifunctional Δ6Δ5 desaturases have been reported in zebrafish *Danio rerio*^28^, rabbitfish *Siganus canaliculatus*^29^, pike silverside *Chirostoma estor*^18^, Nile tilapia *Oreochromis niloticus*^30^, striped snakehead *Channa striata*^10^, African catfish *Clarias gariepinus*^31^, silver barb *Barbonymus gonionotus*^32^ and tambaqui *Collossoma macropomum*^33^. Moreover, Δ4 Fads2, with some activity as Δ5 desaturases, have been reported in rabbitfish^29^, Senegalese sole *Solea senegalensis*^21^, *C*. *estor*^18^, *C*. *striata*^34^, *O*. *niloticus* and Japanese medaka *Oryzias latipes*^35^. Finally, salmonids such as Atlantic salmon *Salmo salar*^36^ and rainbow trout *Oncorhynchus mykiss*^37^ possess Fads2 categorised as monofunctional Δ5 desaturases, although that of salmon was recently reported to also exhibit Δ6 activity^35^.

The precise repertoire and function of teleost Fads2 is species-specific and speculated to be influenced by, or a consequence of, one or more factors including trophic level, habitat (freshwater vs. marine) and trophic ecology^18,21,29^. Among them, the position of the species in the food chain (trophic) was postulated as a major factor influencing LC-PUFA biosynthetic capability in fish^16^ when *S*. *canaliculatus*, a marine herbivore, was found to possess all enzymatic activities required for LC-PUFA biosynthesis^29,38^, an ability previously believed to be restricted to freshwater and salmonid species. To further corroborate whether this hypothesis can be extended to other teleosts, the present study aimed to isolate and molecularly characterise putative *fads2* from four marine teleosts, namely *Sarpa salpa*, *Chelon labrosus*, *Pegusa lascaris* and *Atherina presbyter*, occupying different trophic levels. Where possible, we selected these species because functional data of Fads2 from members of their corresponding phylogenetic groups had been published previously, allowing us clarify whether other factors might also account for functional diversification of teleost Fads2.

## Results

### Sequence and phylogenetic analysis

**Figure 2:**
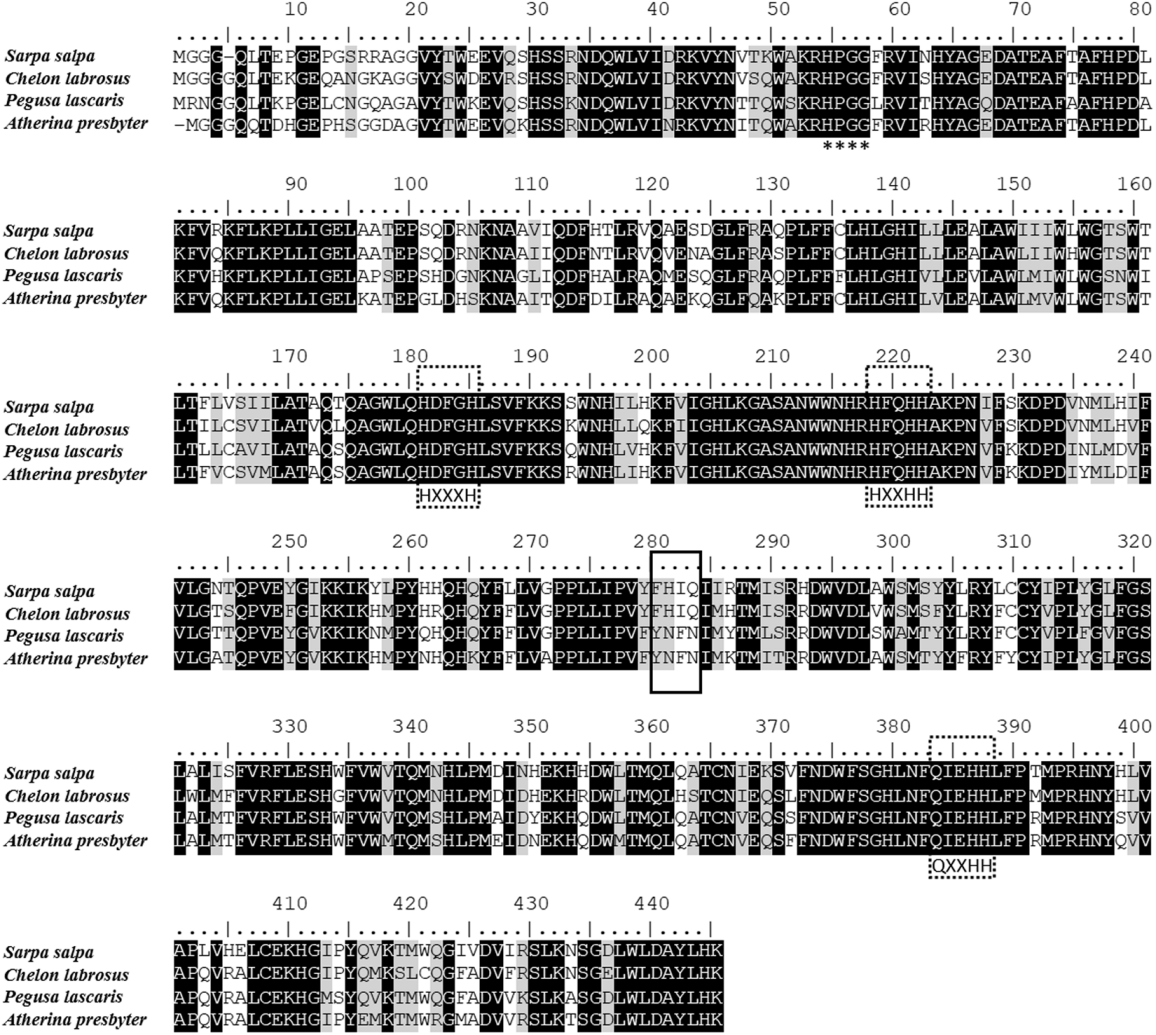
Alignment of the deduced amino acid (aa) sequences of the novel fatty acyl desaturases of *Sarpa salpa*, *Chelon labrosus*, *Pegusa lascaris* and *Atherina presbyter*. Identical residues are shaded black, heme binding motif is indicated by asterisks, histidine boxes are dashed black framed, and regions determining regioselectivity is solid black framed.

The putative *fads2* from *S*. *salpa*, *C*. *labrosus*, *P*. *lascaris* and *A*. *presbyter* all exhibited ORF sequences of 1338 bp encoding putative proteins of 445 amino acids (aa), which were deposited in the GenBank database under the accession numbers MH293506, MH293504, MH293505 and MH293503, respectively. The deduced aa sequences from all species contained typical conserved features of front-end desaturases (Fig. 2), namely a heme binding motif HPGG and three histidine boxes (HXXXH, HXXHH, QXXHH). The four aa regions determining regioselectivity of Fads2 enzymes^39^ varied among the studied species. The *A*. *presbyter* and *P*. *lascaris* Fads2 possessed YXXN aa residues (282–285aa, Fig. 2), while *C*. *labrosus* and *S*. *salpa* Fads2 possessed FXXQ (282–285aa, Fig. 2).

**Figure 3:**
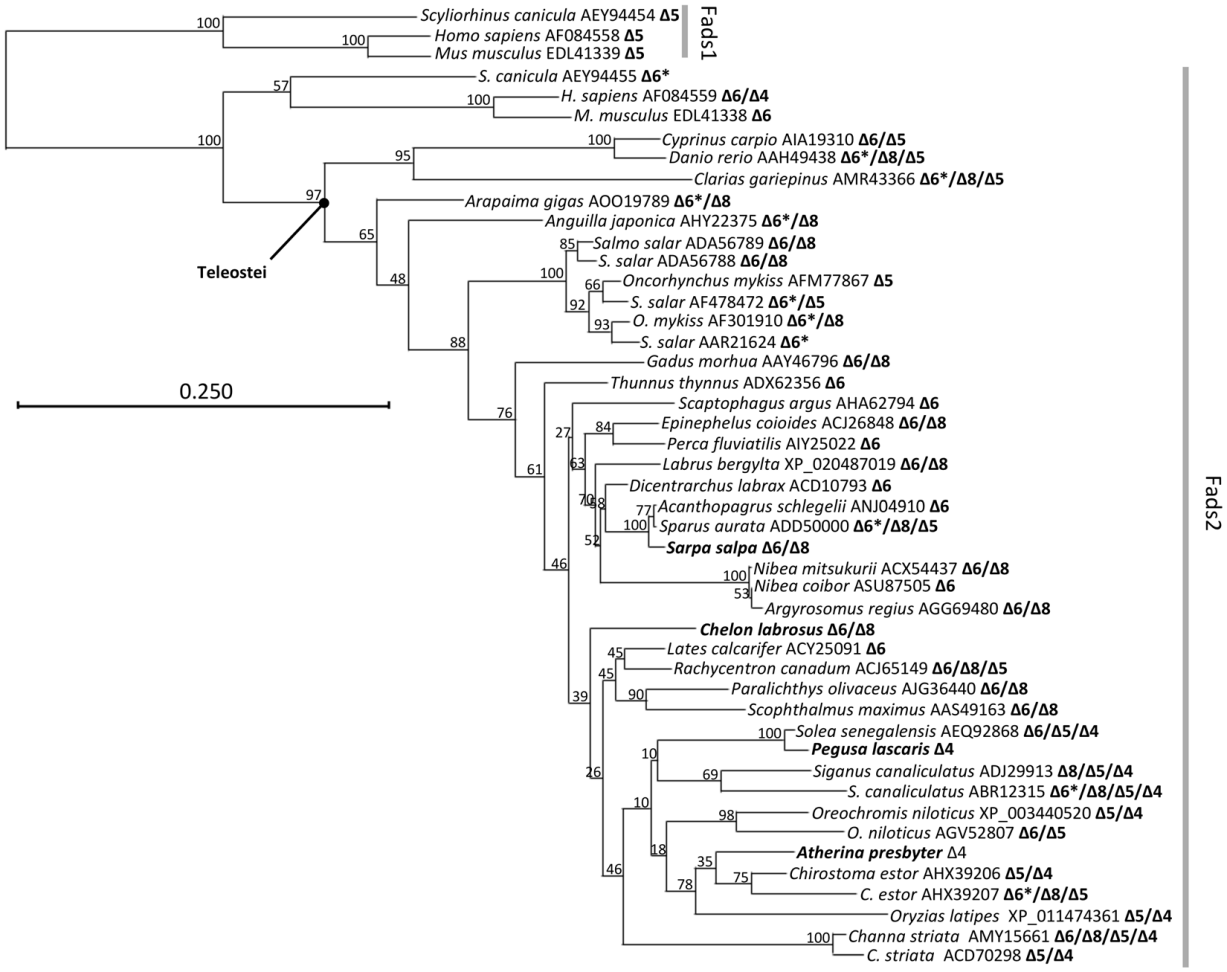
Phylogenetic tree including the deduced amino acid (aa) sequences of the fatty acyl desaturases of *Sarpa salpa*, *Chelon labrosus*, *Pegusa lascaris* and *Atherina presbyter*. The horizontal branch length is proportional to the aa substitution rate per site. Demonstrated desaturase activities are included in all Fads-like sequences as “Δx”. The asterisk (“*”) in some Δ6 desaturases denotes in demonstrated ability of these enzymes to operate towards both C_18_ (e.g. 18:3n-3) and C_24_ (e.g. 24:5n-3) substrates^35^.

The phylogenetic analysis revealed that all putative Fads2 were clustered together with Fads2 from other Teleostei species (bootstrap = 97%), and clearly separated from a branch containing Fads1 sequences (Fig. 3). The *A*. *presbyter* Fads2 was clustered with Atherinomorphae species including Δ4 Fads2 from *O*. *latipes* (XP_011474361) and *C*. *estor* (AHX39206), and Δ6Δ5 Fads2 from *C*. *estor* (AHX39207) (bootstrap = 78%) (Fig. 3). The *S*. *salpa* Fads2 grouped with Δ6 Fads2 from other Sparidae species, namely *Sparus aurata* (ADD50000) and *Acanthopagrus schlegelii* (ANJ04910) (bootstrap = 100%) (Fig. 3). The *P*. *lascaris* Fads2 and *S*. *senegalensis* Δ4 Fads2 (AEQ. 92868) were clustered together (bootstrap = 100%) but this clade was separated from other Pleuronectiformes Δ6 Fads2 such as those of *Scophthalmus maximus* (AAS49163) and *Paralichthys olivaceus* (AJG36440) (Fig. 3).

### Functional characterisation of Fads2 desaturases

Our results show that Fads2 from *S*. *salpa* and *C*. *labrosus* showed Δ6 desaturase activity towards 18:2n-6 and 18:3n-3 since they were able to produce 18:3n-6 and 18:4n-3, respectively, when expressed in yeast (Table 1). Moreover, both *S*. *salpa* and *C*. *labrosus* Fads2 also exhibited Δ8 activity towards, with 20:2n-6 and 20:3n-3 being desaturated to 20:3n-6 and 20:4n-3, respectively (Table 1). No Δ5 or Δ4 desaturations were detected in yeast expressing the *S*. *salpa* and *C*. *labrosus fads2* desaturases (Table 1). Moreover, transgenic yeast expressing *A*. *presbyter* and *P*. *lascaris fads2* showed Δ4 desaturase activity towards 22:4n-6 and 22:5n-3 since they could be desaturated to 22:5n-6 and 22:6n-3, respectively (Table 1). Neither Δ6, Δ8 nor Δ5 desaturase activities were detected in yeast expressing *fads2* from *A*. *presbyter* and *P*. *lascaris* (Table 1).

**Table 1:** Fatty acid conversions in transgenic yeast (*Saccharomyces cerevisiae*) transformed with the Fads2 fatty acyl desaturases of *Sarpa salpa*, *Chelon labrosus*, *Pegusa lascaris* and *Atherina presbyter* grown in the presence of exogenously added fatty acid substrates (18:2n-6, 18:3n-3, 20:2n-6, 20:3n-3, 20:3n-6, 20:4n-3, 22:4n-6 and 22:5n-3).

FA	FA	Conversion (%)				Activity
substrate	product	*S*. *salpa*	*C*. *labrosus*	*P*. *lascaris*	*A*. *presbyter*	
18:2n-6	18:3n-6	9.0	22.4	nd	nd	Δ6
18:3n-3	18:4n-3	19.9	5.5	nd	nd	Δ6
20:2n-6	20:3n-6	2.1	2.3	nd	nd	Δ8
20:3n-3	20:4n-3	3.3	5.7	nd	nd	Δ8
20:3n-6	20:4n-6	nd	nd	nd	nd	Δ5
20:4n-3	20:5n-3	nd	nd	nd	nd	Δ5
22:4n-6	22:5n-6	nd	nd	5.5	5.0	Δ4
22:5n-3	22:6n-3	nd	nd	19.4	11.4	Δ4

Desaturation conversions were estimated by the proportion of substrate fatty acid (FA) converted to desaturated products as [product area/(product area + substrate area)] × 100.

nd, not detected.

### Tissue expression of *fads2*

**Figure 4:**
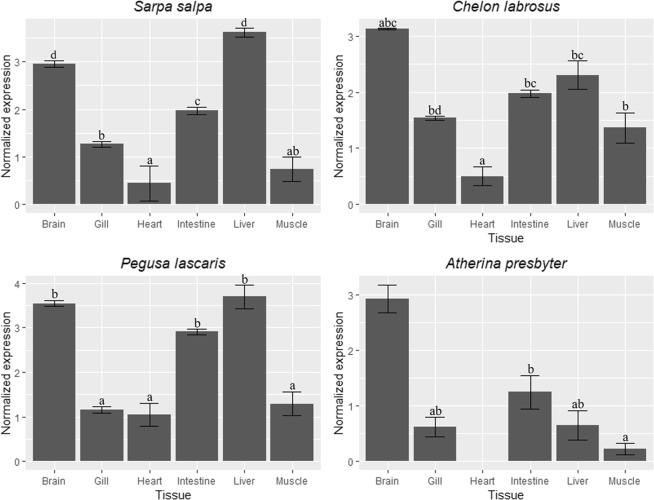
Distribution of *fads2* mRNA levels in tissues of *Sarpa salpa*, *Chelon labrosus*, *Pegusa lascaris* and *Atherina presbyter*. Data are shown as geometric mean log normalised expression ratios ± standard errors (n = 4, except for heart and muscle of *S*. *salpa* where n = 3, brain of *C*. *labrosus* where n = 3, and brain and heart of *A*. *presbyter* where n = 2 and n = 1, respectively). Different letters denote significant differences among tissue within each species (P < 0.05, One-way ANOVA, Tukey HSD test).

Tissue expression of *fads2* in brain, gill, intestine, heart, liver and muscle of the studied species is shown in Fig. 4. Expression of *fads2* was found in all tissues analysed. *S*. *salpa* and *P*. *lascaris* showed the highest *fads2* expression levels in liver, brain and intestine (Fig. 4). A similar pattern was observed in *C*. *labrosus* and *A*. *prebyter*, although the highest expression in brain could not be confirmed statistically (Fig. 4). Heart showed the lowest levels of *fads2* expression among all tissues considered in the analysis (Fig. 4).

## Discussion

Fish vary in their LC-PUFA biosynthetic capacity that, in turn, determines the essential FA requirements in each species. The species’ trophic level, i.e. the position of an organism in the food chain, was proposed as a major factor influencing LC-PUFA biosynthetic capability in fish^16^ after *S*. *canaliculatus*, a marine herbivore, was found to possess all enzymatic activities required to biosynthesise LC-PUFA from C_18_ PUFA precursors, believed previously to be limited to freshwater and salmonid species^29,38^. To clarify whether this assumption could be extended beyond *S*. *canaliculatus*, four marine fish species occupying different trophic levels were selected and their Fads2, a key enzyme in LC-PUFA biosynthesis in teleosts, were characterised^16^. The results of the present study, summarised in Table 2, suggested that the trophic level of a given species cannot alone account for functionalisation of Fads2 occurring in that particular species and thus enabling a more efficient LC-PUFA biosynthesis.

**Table 2:** Fads2 activities, trophic level and environment (freshwater and marine) from the target species studied herein, namely *Sarpa salpa*, *Chelon labrosus*, *Pegusa lascaris* and *Atherina presbyter*, and their corresponding reference species *Sparus aurata*, *Barbonymus gonionotus*, *Solea senegalensis* and *Chirostoma estor*, respectively.

	Target species				Reference species					
Species	Phylogenetic group	Trophic level	Environment	Activity	Species	Phylogenetic group	Trophic level	Environment	Activity	References
*S*. *salpa*	Sparidae	2.00 ± 0.00	Marine	Δ6, Δ8	*S*. *aurata*	Sparidae	3.70 ± 0.00	Marine	Δ6, Δ8	^26,35,42^
*C*. *labrosus*	Mugilidae	2.60 ± 0.32	Marine	Δ6, Δ8	*B*. *gonionotus*	Cyprinidae	2.40 ± 0.13	Freshwater	Δ6, Δ5, Δ8	^32^
*P*. *lascaris*	Soleidae	3.30 ± 0.10	Marine	Δ4	*S*. *senegalensis*	Soleidae	3.30 ± 0.46	Marine	Δ4	^21^
*A*. *presbyter*	Atherinidae	3.70 ± 0.43	Marine	Δ4	*C*. *estor*	Atherinidae	3.30 ± 0.20	Freshwater	Δ4	^18,35^

Acquision of new or additional functions (neo and subfunctionalisation) within Fads2 is an advantageous feature in animals such as teleosts that have lost Fads1 with Δ5 activity. Possessing such a trait, however, cannot be linked directly to trophic level of the species, but rather appears to be more dependent upon the position that particular species occupies within the teleost clade. Like *S*. *canaliculatus*, the herein studied *S*. *salpa* is a truly herbivorous fish species thriving on low LC-PUFA diets such as *Rhodophyta* sp.^40^ and thus potentially having also high LC-PUFA biosynthetic capacity as its herbivore counterpart *S*. *canaliculatus*^29,38^. Interestingly, *S*. *salpa* was chosen for this study to enable us to compare its Fads2 function with those of the carnivorous Sparidae counterparts, *A*. *schlegelii* and *S*. *aurata* (trophic levels of 3.2 ± 0.45 and 3.7 ± 0.0, respectively) (Table 2), whose diets consist of marine invertebrates that are naturally rich in LC-PUFA^41^. Despite the markedly different trophic levels existing among these Sparidae species, *S*. *salpa* Fads2 was functionally characterised as a Δ6 desaturase like those from *S*. *aurata* and *A*. *schlegelii*^42,43^. These results suggest that, rather than trophic level as we initially hypothesised, phylogeny of the species might be a more important factor influencing Fads2 regioselectivity and, ultimately, the species’ ability for LC-PUFA biosynthesis. In agreement, certain teleost families such as Cichlidae include species with two functionalised Fads2, namely a Δ4 desaturase^35^ and a Δ6Δ5 desaturase^30^, which appear to be present across the entire family^35^. Similarly, the presence of Δ4 Fads2 reported herein in *A*. *presbyter* and *P*. *lascaris* (Table 2), is consistent with this desaturase type also being present in species within their corresponding orders, namely *C*. *estor* (Atheriniformes) and *S*. *senegalensis* (Pleuronectiformes)^18,19,21^. Interestingly, while members of the Soleidae species like *S*. *senegalensis* and *P*. *lascaris* possess Fads2 with Δ4 activity, Scophthalmidae species such as *S*. *maximus* and *P*. *olivaceus* have a Δ6 Fads2^19,42^. While characterisation of further Fads2 from species within other teleost orders is required to establish more fully the distribution of functionalised Fads2 within this vertebrate clade, it is becoming apparent that the occurrence of Fads2 with a non-Δ6 phenotype is more common than initially anticipated, and instances of functionalised Fads2 have occurred in basal teleost lineages such us Osteoglossomorpha^25^.

It is still unclear which aa residues of teleost Fads2 are responsible for the dual Δ6Δ5 desaturase phenotype reported in some species^25^. However, the aa sequence of a particular region in Fads2 located within the second and third histidine boxes has been identified as crucial for the regioselectivity of Δ4 Fads2^35,39^. In the present study, both Δ4 Fads2 characterised from *A*. *presbyter* and *P*. *lascaris* contained the specific aa residues YXXN that account for the Δ4 activity in Fads2 enzymes^35,39^. This is consistent with the pattern found in Δ4 Fads2 from *S*. *canaliculatus*, *S*. *senegalensis*, *O*. *niloticus* and *O*. *latipes*^21,24,26,29,30,35^ but differs from the YXXQ regioselectivity region of the Δ4 Fads2 from *C*. *striata*^10,34^. In contrast, the regioselectivity region of the Fads2 characterised from *S*. *salpa* and *C*. *labrosus* contained FXXQ, which is consistent with other Δ6 Fads2 from *R*. *canadum*, *Gadus morhua*, *S*. *salar* and *Arapaima gigas*^20,44–46^. Interestingly, Fads2 desaturases with the FXXQ sequence also include the bifunctional Δ6Δ5 desaturases from *D*. *rerio*^28^, *S*. *canaliculatus*^29^ and *C*. *striata*^10^ and, thus, this sequence is not useful to predict function of teleost Fads2.

Monroig *et al*.^26^ established that Δ8 desaturase capacity is an intrinsic feature among teleost Fads2. We herein investigated the ability of the Fads2 for Δ8 desaturation and concluded that Δ6 Fads2 from *S*. *salpa* and *C*. *labrosus* were also Δ8 desaturases. These results are in agreement with virtually all Fads2 with Δ6 or Δ6Δ5 desaturation capabilities also having Δ8 activity^10,18,19,25,26,31,33^. In contrast, Fads2 from *P*. *lascaris* and *A*. *presbyter*, characterised as Δ4 desaturases in the present study, did not show Δ8 desaturase activity, consistent with such an ability also being absent in Δ4 Fads2 from *C*. *estor*^18^ and *C*. *striata*^34^. Crystalline structure is available for two mammalian stearoyl-CoA desaturases^47,48^ but, unfortunately, is lacking for Fads-like desaturases such as those studied herein. Therefore, it is not yet possible to understand the catalytic mechanism by which Δ8 desaturation ability is retained in some Fads2, but lost in neofunctionalised Δ4 Fads2. Additionally, from a metabolic standpoint, it is difficult to explain what advantage might be associated with having Fads2 enabling two alternative routes (i.e. Δ6 and Δ8 pathways), but which do not overcome the lack of Δ5 desaturation capacity required for EPA and ARA biosynthesis. Indeed, Δ5 desaturase appears to also be limiting the LC-PUFA biosynthetic capacity in *P*. *lascaris* and *A*. *presbyter* that, despite having Δ4 desaturases, did not show any Δ5 desaturase activity. All the implications discussed herein should be taken with caution considering that other Fads2 might exist within these species’ genomes. Further studies are required to confirm the presence and functions of further Fads2 with potentially complementary roles to those of the Fads2 characterised in the present study.

The analysis of *fads2* tissue expression showed significant differences within each species. The four species are all of marine origin, with two out of the four (*S*. *salpa* and *P*. *lascaris*) displaying the highest number of mRNA copies in brain and liver and also in intestine from *P*. *lascaris*. This is generally in agreement with these tissues being major metabolic sites for LC-PUFA biosynthesis in fish^19,30,31,37,45,46,49–51^. In general, freshwater fish species have been shown to express highest *fads2* expression levels in liver and intestine^10,17,18,52^, with marine species often having brain as the tissue with the highest expression^17^. Nevertheless, this is a broad generalisation since, in addition to habitat (freshwater vs. marine), other factors such as developmental stage, nutritional history, reproductive status, etc., can influence the tissue distribution patterns of fish *fads2*^17^. Unfortunately, the impact that these factors might have had on the specimens used in our tissue distribution analyses could not be determined, and thus we are unable to clarify whether any correlations exist between our expression data and the species’ trophic level.

In conclusion, the data indicated that trophic level does not appear to be the primary determinant of Fads2 activity and does not, by itself, explain cases of functionalisation within the Fads2 protein family. In the herbivorous Sparidae species *S*. *salpa*, the Fads2 functions were found to be very similar to those previously reported in Fads2 from carnivorous counterpart species such as *S*. *aurata* and *A*. *schlegelii*. Instances of functionalised Fads2 with Δ4 desaturase activity enabling enhanced LC-PUFA biosynthesis were found in higher trophic level species, despite the fact that the essential FA requirements of these species can likely be fully satisfied by their natural diets.

## Methods

This study was conducted in accordance with the regulations within Spanish law 6/2013 based on Directive 2010/63/EU of the European Parliament on animal welfare and the protection of animals used for scientific purposes. The experiment was authorised by the Ethics Committee of University of La Laguna (Spain) and the Animal Welfare and Ethical Review Board of University of Stirling (UK).

### Fish and tissue collection

Four individual specimens of salema (*S*. *salpa*), thicklip grey mullet *(C*. *labrosus*), sand sole (*P*. *lascaris*) and sand smelt (*A*. *presbyter*), were net caught by local fishermen. These species occupy different trophic levels: 2.0 ± 0.0 (herbivore, *S*. *salpa*), 2.6 ± 0.32 (omnivore, *C*. *labrosus*), 3.3 ± 0.1 (carnivore, *P*. *lascaris*) and 3.7 ± 0.4 (carnivore, *A*. *presbyter*)^41^. The *S*. *salpa*, *C*. *labrosus*, and *A*. *presbyter* specimens were captured off the coast of Tenerife (Spain), whereas *P*. *lascaris* was captured off the coast of Huelva (Spain) (Supplementary Table S1). Fish were killed after anesthesia by percussive blow to the head prior to collection of tissues including brain, gill, intestine (specifically foregut), heart, liver and muscle. The tissue samples (~100 mg for *S*. *salpa*, *C*. *labrosus* and *P*. *lascaris*, and ~50 mg for *A*. *presbyter*) were immediately placed in tubes containing RNA*later* (Qiagen Iberia S.L., Spain), and maintained at 4 °C for 24 h prior to being frozen at −20 °C until analysis.

### Molecular cloning of fads2 cDNAs

Total RNA was extracted from each tissue of each species using TRI Reagent (Sigma-Aldrich, UK) according to the manufacturer’s instructions using a bead tissue disruptor (Bio Spec, USA). For each species, first-strand complementary DNA (cDNA) was synthesised from 2 µg of total RNA from brain and liver (1:1 mixture) using High Capacity cDNA Reverse Transcription Kit (AB Applied Biosystems, USA). In order to obtain the first fragments of *fads2* genes by polymerase chain reaction (PCR), the cDNA was used as template together with degenerated primers (Supplementary Table S2) and GoTaq^®^ Green Master Mix (Promega, UK). Degenerate primers were designed on conserved regions of teleost *fads2* sequences obtained using the NCBI blastn tool (http://www.ncbi.nlm.nih.gov/), specifically *G*. *morhua* (DQ054840.2), *S*. *senegalensis* (JN673546.1), *S*. *aurata* (AY055749.1), *Epinephelus coioides* (EU715405.1), *Rachycentron canadum* (FJ440238.1), *S*. *canaliculatus* (EF424276.2), and *C*. *estor* (KJ417838.1 and KJ417839.1), which were then aligned with BioEdit v7.0.9 (Tom Hall, Department of Microbiology, North Carolina State University, USA). PCR were performed by an initial denaturing step at 95 °C for 2 min, followed by 35 cycles of denaturation at 95 °C for 30 s, annealing at 58–62 °C for 30 s and extension at 72 °C for 1 min and 30 s, followed by a final extension at 72 °C for 5 min (Supplementary Table S3). The PCR fragments were purified on agarose gels using Illustra^TM^ GFX^TM^ PCR DNA and Gel Band Purification kit (GE Healthcare Life Sciences, UK), cloned into pGEM-T Easy vector (Promega, UK) and sequenced (GATC Biotech, Germany).

The obtained sequences were used to design specific primers for 5′ and 3′ Rapid Amplification of cDNA Ends (RACE) PCR to obtain full-length cDNA sequences of *fads2* (FirstChoice^®^ RLM-RACE kit, Ambion, Applied Biosystems, UK). Details of all primers used for RACE PCR are provided in Supplementary Table S2. For 3′RACE PCR, a positive fragment was obtained for *fads2* by two-round PCR (see conditions in Supplementary Table S3). In the first round PCR, 3′RACE outer primer and gene-specific primers for each species were used, using 3′RACE cDNA as a template. In the second round, the first-round product of the putative *fads2* from each species was used as a template for nested PCR along with 3′RACE inner primer and gene-specific primers. For 5′RACE PCR (Supplementary Table S3), a similar procedure was followed, with the first round PCR using 5′RACE cDNA as template run with 5′RACE outer primer and gene-specific primers (Supplementary Table S2). The second round PCR used 5′RACE inner and gene-specific primers (Supplementary Table S2) and the first round RACE PCR products as a template (see conditions in Supplementary Table S3). Both 3′ and 5′ RACE PCR fragments of each species’ *fads2* were purified, cloned and sequenced as described above.

### Sequence and phylogenetic analyses

The deduced amino acid (aa) sequences of putative Fads2 proteins isolated from *A*. *presbyter*, *C*. *labrosus*, *P*. *lascaris* and *S*. *salpa* were aligned with multiple functionally characterised Fads2 desaturases using MAFFT (https://mafft.cbrc.jp/alignment/software/) Ver. 7.388^53^ with the G-INS-I method. The obtained alignment was then cropped to remove columns containing gaps in 95% or more of the sequences. The maximum likelihood (ML) phylogenetic analysis was performed using PhyML v3.0 server^54^. The evolutionary model of protein used for constructing the tree was JTT + G + I, which was selected by Smart Model Selection (SMS) with Bayesian information criterion^55^ (BIC). The branch supporting values were calculated from 1000 bootstrap replicates. The resultant ML tree was visualised using CLC Main Workbench 8.0 (CLC bio, Denmark).

### Functional characterisation of the Fads2 by heterologous expression in Saccharomyces cerevisiae

The open reading frames (ORF) of the *fads2* cloned from *A*. *presbyter*, *C*. *labrosus*, *P*. *lascaris* and *S*. *salpa* were amplified from liver cDNA using a nested PCR approach. The first round PCR was run using primer pairs designed in the 5′ and 3′ untranslated regions (UTR) for forward and reverse primers, respectively (Supplementary Table S2). The second round PCR was run using the first round PCR products as templates and primers containing the restriction enzymes *Bam*HI/*Xho*I for *A*. *presbyter* and *P*. *lascaris fads2*, and *Hin*dIII/*Xho*I for *C*. *labrosus* and *S*. *salpa fads2* (underlined in Supplementary Table S2). Both first and second round PCR were performed with the high fidelity *Pfu* DNA polymerase (Promega, UK) for *fads2* from *A*. *presbyter*, *C*. *labrosus* and *P*. *lascaris*, and PfuUltra II Fusion HS DNA Polymerase (Agilent, USA) for *S*. *salpa fads2*. Further details on PCR conditions used to amplify *fads2* ORF sequences are given in Supplementary Table S3. The PCR products were subsequently purified as described above, digested with the corresponding restriction enzymes (New England BioLabs, UK) and ligated into a similarly restricted pYES2 yeast expression vector (Thermo Fisher Scientific, UK). The plasmids containing pYES2-*fads2* from each species were sequenced before being transformed into yeast *Saccharomyces cerevisiae* competent cells InvSc1 (Thermo Fisher Scientific).

Transformation and selection of yeast culture were performed as described previously^20,56^. One single yeast colony transformed with pYES2-*fads2* for each species was used in each functional assay. The transgenic yeasts were grown in the presence of FA substrates for Δ6 (18:2n-6 and 18:3n-3), Δ8 (20:2n-6 and 20:3n-3), Δ5 (20:3n-6 and 20:4n-3) and Δ4 (22:4n-6 and 22:5n-3) desaturases. The FA substrates were added to the yeast cultures at final concentrations of 0.5 mM for C_18_ PUFA 0.75 mM for C_20_ PUFA and 1.0 mM for C_22_ PUFA, as uptake efficiency decreases with increasing chain length^44^. In addition, yeasts transformed with empty pYES2 were also grown in the presence of each substrate as control treatments. After 2 days of culture at 30 °C, yeasts were harvest, washed, and total lipid extracted by homogenisation in chloroform/methanol (2:1, v/v) containing 0.01% (w/v) butylated hydroxytoluene (BHT) as antioxidant.

### Fatty acid analysis of yeast

Fatty acid methyl esters (FAME) were prepared from total lipid extracted from yeast according to Hastings *et al*.^28^. FAME were separated and quantified using a Fisons GC-8160 (Thermo Fisher Scientific) gas chromatograph equipped with a 60 m × 0.32 mm i.d. × 0.25 μm ZB-wax column (Phenomenex, UK) and flame ionisation detector^57^. The desaturation conversion efficiencies from exogenously added PUFA substrates were calculated by the proportion of substrate fatty acid converted to desaturated products as [product area/(product area + substrate area)] × 100.

### Tissue expression of fads2 in each species

Expression of the *fads2* genes was determined by quantitative real-time PCR (qPCR) in brain, gill, intestine, heart, liver and muscle. Replicate numbers were n = 4 in each species except for brain of *C*. *labrosus* and *A*. *presbyter* (n = 3 and n = 2, respectively), heart of *S*. *salpa* and *A*. *presbyter* (n = 3 and n = 1, respectively) and muscle of *S*. *salpa* (n = 3). Elongation factor-1α (*elf1α*) and *β-actin* were used as reference genes to normalise the expression of *fads2*. For each tissue 2 µg of total RNA were reverse transcribed into cDNA as described above. In order to determine the efficiency of the primer pairs, serial dilutions of pooled cDNA from all four tissues were carried out. All qPCR were performed by a Biometra TOptical Thermocycler (Analytik Jena, Germany) in 96-well plates in duplicates at total volume of 20 µL containing 10 µL of Luminaris Color HiGreen qPCR Master Mix (Thermo Fisher Scientific), 1 µL of each primer (10 µM), 2 µL or 5 µL of cDNA (1/20 dilutions) for reference and target genes respectively, as well as 6 or 3 µL of molecular biology grade water. Besides, negative controls (NTC, no template control), containing molecular biology grade water instead of cDNA, were also run. The primer sequences and qPCR conditions are detailed in Supplementary Tables S2 and S3, respectively. The relative expression of *fads2* among tissues in each species was calculated as arbitrary units after normalisation dividing by the geometric mean of the expression level of the reference genes *elf1α* and *β-actin*. One arbitrary unit is defined as the ratio between the expression level of *fads2* and the lowest expression level for this gene. After each qPCR analysis, a melting curve with 1 °C increments during 6 s from 60 to 95 °C was performed, in order to check the presence of a single product in each reaction.

### Statistical analysis

Tissue expression results are presented as log 10 mean normalised ratios ± standard error. Data were checked for normal distribution with the one-sample Shapiro-Wilk test, as well as for homogeneity of the variances with the Levene’s test^58^. A one-way ANOVA test was performed within each species for tissue expression factor, followed by a Tukey HSD multiple comparison test^58^. When normal distribution and/or homoscedasticity were not achieved, data were subjected to Kruskall-Wallis non-parametric test, followed by Pairwise Wilcoxon Rank Sum Tests^58^. Statistical analysis was carried out in tissues from at least three replicates, and their significance was established at P < 0.05. Statistical analyses were performed using the R software (R Core Team, Austria).

## Supplementary information

Functional diversification of teleost Fads2 fatty acyl desaturases occurs independently of the trophic level
