# Supplementary material for: Functional diversification of teleost Fads2 fatty acyl desaturases occurs independently of the trophic level
Source: Sci Rep. 2019 Aug 1;9:11199. doi: 10.1038/s41598-019-47709-0 (PMC6671994; doi:10.1038/s41598-019-47709-0)
Supplement: Supplementary file 1 — Functional diversification of teleost Fads2 fatty acyl desaturases occurs independently of the trophic level [file 41598_2019_47709_MOESM1_ESM.docx]

**Functional diversification of teleost Fads2 fatty acyl desaturases occurs independently of the trophic level**

Diego Garrido^1^, Naoki Kabeya^2^, Mónica B. Betancor^3^, José A. Pérez^1^, N. Guadalupe Acosta^1^, Douglas R. Tocher^3^, Covadonga Rodríguez^1^*, Óscar Monroig^4^*

^1^ Departamento de Biología Animal, Edafología y Geología, Universidad de La Laguna, La Laguna 38206, Santa Cruz de Tenerife, Spain (diegogarridolorenzo@gmail.com; janperez@ull.es; ngacostaglez@gmail.com; covarodr@ull.edu.es)

^2^ Department of Aquatic Bioscience, The University of Tokyo, Yayoi, Bunkyo-ku, Tokyo, Japan (naokikby@g.ecc.u-tokyo.ac.jp)

^3^ Institute of Aquaculture, Faculty of Natural Sciences, University of Stirling, Stirling FK9 4LA, Scotland, UK (m.b.betancor@stir.ac.uk; d.r.tocher@stir.ac.uk)

^4^ Instituto de Acuicultura Torre de la Sal, Consejo Superior de Investigaciones Científicas (IATS-CSIC), 12595 Ribera de Cabanes, Castellón, Spain (oscar.monroig@csic.es)

*Corresponding equally to this work.

Correspondence to: oscar.monroig@csic.es; covarodr@ull.edu.es

**Supplementary Table S1.** Fresh weight of specimens of *Sarpa salpa*, *Chelon labrosus*, *Pegusa lascaris* and *Atherina presbyter.*

| Species | Fresh weight (g) | | |
| --- | --- | --- | --- |
| *S. salpa* | 93.7 | ± | 2.5 |
| *C. labrosus* | 46.6 | ± | 15.4 |
| *P. lascaris* | 112.0 | ± | 18.9 |
| *A. presbyter* | 1.6 | ± | 0.3 |

**Supplementary Table S2.** Sequences of primer pairs used in the cloning of the *Sarpa salpa*, *Chelon labrosus*, *Pegusa lascaris*, and *Atherina presbyter* fatty acyl desaturase (*fads2*) ORF and for quantitative real-time PCR (qPCR) analysis of gene expression in tissues. Accession numbers of the sequences used as references for primer design are also detailed. Restriction sites *Bam*HI/*Xho*I for *A. presbyter* (APFads2VF/APFads2VR) and *P. lascaris* (PLFads2VF/PLFads2VR) and *Hin*dIII/*Xho*I for *C. labrosus* (CLFads2VF/CLFads2VR) and *S. salpa* (SSFads2VF/SSFads2VR) are underlined in the corresponding primer sequences.

| **Aim** | **Species** | **Transcript** | **Primers** | **Primers sequence** |
| --- | --- | --- | --- | --- |
| First Fragment | S. salpa | fads2 | FFFads2F1 | 5’-TACACCTGGGAGGAGGTGCAG-3’ |
|  |  |  | FFFads2R1 | 5’-TGTCCGCTGAACCAGTCGTTGAA-3’ |
|  | *C. labrosus* |  | FFFads2F2 | 5’-TACACCTGGRAGGAGGTGCAG-3’ |
|  |  |  | FFFads2R1 | 5’-TGTCCGCTGAACCAGTCGTTGAA-3’ |
|  | *P. lascaris* |  | FFFads2F3 | 5’-TACACCTGGRAGGAGGTGCAG-3’ |
|  |  |  | FFFads2R2 | 5’-TGTCCRCTGAACCAGTCGTTGAA-3’ |
|  | *A. presbyter* |  | FFFads2F1 | 5’-TACACCTGGGAGGAGGTGCAG-3’ |
|  |  |  | FFFads2R1 | 5’-TGTCCGCTGAACCAGTCGTTGAA-3’ |
| RACE PCR | *S. salpa* | *fads2* | 3’SSFads2F1 | 5’-GCTCAGCCGTTGTTCTTCTG-3’ |
|  |  |  | 3’SSFads2F2 | 5’-TCCAGCATCACGCTAAACCC-3’ |
|  |  |  | 5’SSFads2R1 | 5’-CTCTCCGCCTGCACACGTAA-3’ |
|  |  |  | 5’SSFads2R2 | 5’-GGGGTGAAAAGCAGTGAACG-3’ |
|  | *C. labrosus* |  | 3’CLFads2F1 | 5’-GCCAACTGGTGGAATCATCG-3’ |
|  |  |  | 3’CLFads2F2 | 5’-TCCAACATCATGCTAAACCC-3’ |
|  |  |  | 5’CLFads2R1 | 5’-CTGTACTCGCAGGGTGTTGA-3’ |
|  |  |  | 5’CLFads2R2 | 5’-TTTTGGCCCACTGAGAGACG-3’ |
|  | *P. lascaris* |  | 3’PLFads2F1 | 5’-GATTGGAGAGCTGGCACCAT-3’ |
|  |  |  | 3’PLFads2F2 | 5’- CACAGCCTGTTGAGTACGGT-3’ |
|  |  |  | 5’PLFads2R1 | 5’-GCGAGGACCTCTAGCAACAC-3’ |
|  |  |  | 5’PLFads2R2 | 5’- TGAGCTCGAAACAGACCCTG-3’ |
|  | *A. presbyter* |  | 3’APFads2F1 | 5’-TTTTGGAGAGCCACTGGTTTGT-3’ |
|  |  |  | 3’APFads2F2 | 5’-GAGAAGCACCAAGACTGGATGA-3’ |
|  |  |  | 5’APFads2R1 | 5’-AAGCAGTGAAAGCTTCCGTGG-3’ |
|  |  |  | 5’APFads2R2 | 5’-CCAGCATAGTGGGCGGATG-3’ |
| ORF cloning | *S. salpa* | *fads2* | SSFads2U5F | 5’-GGTGGATCCAGACCAGAGACAGCGCT-3’ |
|  |  |  | SSFads2U3R | 5’-CTCCTTCAAGGTACAGAAAGAGAAG-3’ |
|  |  |  | SSFads2VF | 5’-CCCAAGCTTAGGATGGGAGGTGGAGG-3’ |
|  |  |  | SSFads2VR | 5’-CCGCTCGAGTCATTTATGGAGATAAGCATCGAGCC-3’ |
|  | *C. labrosus* | *fads2* | CLFads2U5F | 5’-CGTCGAATGATCTGCTCGGACTTGATCC-3’ |
|  |  |  | CLFads2U3R | 5’-GTAACGTCACTCCTTTCGCA-3’ |
|  |  |  | CLFads2VF | 5’-CCCAAGCTTAGGATGGGAGGCG-3’ |
|  |  |  | CLFads2VR | 5’-CCGCTCGAGTTATTTATGGAGATATGC-3’ |
|  | *P. lascaris* | *fads2* | PLFads2U5F | 5’- ACACAGGAACAGCCGGGAGGAG-3’ |
|  |  |  | PLFads2U3R | 5’- GTGTGTGTGTGCGTGTGTGGGATAGAAA-3’ |
|  |  |  | PLFads2VF | 5’- CCCGGATCCAGGATGAGAAACGGAGGCCAGCTG-3’ |
|  |  |  | PLFads2VR | 5’- CCGCTCGAGTCATTTATGGAGATATGCATCAAGCCAGAGG-3’ |
|  | *A. presbyter* | *fads2* | APFads2U5F | 5’-GGATTCCGGTCAGACTCAGCAGCAG-3’ |
|  |  |  | APFads2U3R | 5’-CATTTCTCATTTCACAGGGACG-3’ |
|  |  |  | APFads2VF | 5’-CCCGGATCCAGGATGGGAGGTGGA-3’ |
|  |  |  | APFads2VR | 5’-CCGCTCGAGTCATTTATGAAGATATGC-3’ |
| qPCR | *S. salpa* | *fads2* | SSFasd2qF1 | 5’-GTCCGTCTTCAACGACTGGT-3’ |
|  |  |  | SSFasd2qR1 | 5’-GATAACGTCGACGATGCCCT-3’ |
|  | *C. labrosus* |  | CLFasd2qF1 | 5’-GTGTCAAGGCTTCGCTGATG-3’ |
|  |  |  | CLFasd2qR1 | 5’-AACGTCACTCCTTTCGCATACA-3’ |
|  | *P. lascaris* |  | PLFasd2qF1 | 5’-GTTTCCTAGGATGCCCCGCC-3’ |
|  |  |  | PLFasd2qR1 | 5’-TGGAGATATGCATCAAGCCAGA-3’ |
|  | *A. presbyter* |  | APFasd2qF1 | 5’-AGTCTCGGTGGAATCACCTG-3’ |
|  |  |  | APFasd2qR1 | 5’-ACTGGCTGAGTGGCTCCTAA-3’ |
|  | *S. salpa* | *β-actin* | β*-actin*qF1 | 5’-CAGGGAGAAGATGACCCAGA-3’ |
|  |  |  | β*-actin*qR1 | 5’-ACAGTGCCCATCTATGAGGG-3’ |
|  | *C. labrosus* |  | β*-actin*qF2 | 5’-CAGGGAGAAGATGACCCAGA-3’ |
|  |  |  | β*-actin*qR2 | 5’-CCCTCGTAGATGGGCACTGT-3’ |
|  | *P. lascaris* |  | β*-actin*qF1 | 5’-CAGGGAGAAGATGACCCAGA-3’ |
|  |  |  | β*-actin*qR1 | 5’-ACAGTGCCCATCTATGAGGG-3’ |
|  | *A. presbyter* |  | β*-actin*qF2 | 5’-CAGGGAGAAGATGACCCAGA-3’ |
|  |  |  | β*-actin*qR2 | 5’-CCCTCGTAGATGGGCACTGT-3’ |
|  | *S. salpa* | *elf1α* | *efl1α*qF1 | 5’-ATGCACCACGAGTCTCTGAC-3’ |
|  |  |  | *efl1α*qR1 | 5’-GGGTGGTTCAGGATGATGAC-3’ |
|  | *C. labrosus* |  | *efl1α*qF2 | 5’-GTCGAGATGCACCACGAGTC-3’ |
|  |  |  | *efl1α*qR1 | 5’-GGGTGGTTCAGGATGATGAC-3’ |
|  | *P. lascaris* |  | *efl1α*qF3 | 5’-GTGGAGATGCACCACGAGTC-3’ |
|  |  |  | *efl1α*qR1 | 5’-GGGTGGTTCAGGATGATGAC-3’ |
|  | *A. presbyter* |  | *efl1α*qF3 | 5’-GTGGAGATGCACCACGAGTC-3’ |
|  |  |  | *efl1α*qR1 | 5’-GGGTGGTTCAGGATGATGAC-3’ |

**Supplementary Table S3.** Reaction conditions for cloning, functional characterisation and gene expression of *fads2* in *Sarpa salpa*, *Chelon labrosus*, *Pegusa lascaris* and *Atherina presbyter.*

| **Aim** | **Species** | **Transcript** | **Forward primer** | **Reverse primer** | **Denaturing temperature (°C) (duration in s)** | **Annealing temperature (°C) (duration in s)** | **Extension temperature (°C) (duration in s)** | **Number of cycles** |
| --- | --- | --- | --- | --- | --- | --- | --- | --- |
| First Fragment | *S. salpa* | *fads2* | FFFads2F1 | FFFads2R1 | 95 (30) | 62 (30) | 72 (90) | 35 |
|  | *C. labrosus* |  | FFFads2F2 | FFFads2R1 | “ | 58 (30) | “ | “ |
|  | *P. lascaris* |  | FFFads2F3 | FFFads2R2 | “ | 62 (30) | “ | “ |
|  | *A. presbyter* |  | FFFads2F1 | FFFads2R1 | “ | “ | “ | “ |
| RACE PCR | *S. salpa* | *fads2* | 5' RACE Outer | 5’SSFads2R1 | 95 (30) | 58 (30) | 72 (90) | 35 |
|  |  |  | 5' RACE Inner | 5’SSFads2R2 | “ | “ | “ | “ |
|  |  |  | 3’SSFads2F1 | 3' RACE Outer | “ | “ | “ | “ |
|  |  |  | 3’SSFads2F2 | 3' RACE Inner | “ | “ | “ | “ |
|  | *C. labrosus* |  | 5' RACE Outer | 5’CLFads2R1 | “ | “ | “ | “ |
|  |  |  | 5' RACE Inner | 5’CLFads2R2 | “ | “ | “ | “ |
|  |  |  | 3’CLFads2F1 | 3' RACE Outer | “ | “ | “ | “ |
|  |  |  | 3’CLFads2F2 | 3' RACE Inner | “ | 59 (30) | “ | “ |
|  | *P. lascaris* |  | 5' RACE Outer | 5’PLFads2R1 | “ | 58 (30) | “ | “ |
|  |  |  | 5' RACE Inner | 5’PLFads2R2 | “ | “ | “ | “ |
|  |  |  | 3’PLFads2F1 | 3' RACE Outer | “ | “ | “ | “ |
|  |  |  | 3’PLFads2F2 | 3' RACE Inner | “ | 59 (30) | “ | “ |
|  | *A. presbyter* |  | 5' RACE Outer | 5’APFads2R1 | “ | 58 (30) | “ | “ |
|  |  |  | 5' RACE Inner | 5’APFads2R2 | “ | “ | “ | “ |
|  |  |  | 3’APFads2F1 | 3' RACE Outer | “ | “ | “ | “ |
|  |  |  | 3’APFads2F2 | 3' RACE Inner | “ | 59 (30) | “ | “ |
| ORF cloning | *S. salpa* | *fads2* | SSFads2U5F | SSFads2U3R | 95 (20) | 65 (20) | 72 (105) | 40 |
|  |  |  | SSFads2VF | SSFads2VR | “ | “ | “ | “ |
|  | *C. labrosus* | *fads2* | CLFads2U5F | CLFads2U3R | 95 (30) | 57 (30) | 72 (180) | “ |
|  |  |  | CLFads2VF | CLFads2VR | “ | 60 (30) | “ | “ |
|  | *P. lascaris* | *fads2* | PLFads2U5F | PLFads2U3R | “ | “ | 72 (210) | 35 |
|  |  |  | PLFads2VF | PLFads2VR | “ | “ | “ | “ |
|  | *A. presbyter* | *fads2* | APFads2U5F | APFads2U3R | “ | 58 (30) | “ | “ |
|  |  |  | APFads2VF | APFads2VR | “ | 61 (30) | “ | “ |
| qPCR | *S. salpa* | *fads2* | SSFasd2qF1 | SSFasd2qR1 | 95 (15) | 58.5 (30) | 72 (30) | 35 |
|  | *C. labrosus* |  | CLFasd2qF1 | CLFasd2qR1 | “ | “ | “ | “ |
|  | *P. lascaris* |  | PLFasd2qF1 | PLFasd2qR1 | “ | “ | “ | “ |
|  | *A. presbyter* |  | APFasd2qF1 | APFasd2qR1 | “ | “ | “ | “ |
|  | *S. salpa* | *β-actin* | β*-actin*qF1 | β*-actin*qR1 | 95 (15) | 58.5 (30) | 72 (30) | 35 |
|  | *C. labrosus* |  | β*-actin*qF2 | β*-actin*qR2 | “ | “ | “ | “ |
|  | *P. lascaris* |  | β*-actin*qF1 | β*-actin*qR1 | “ | “ | “ | “ |
|  | *A. presbyter* |  | β*-actin*qF2 | β*-actin*qR2 | “ | “ | “ | “ |
|  | *S. salpa* | *elf1α* | *efl1α*qF1 | *efl1α*qR1 | 95 (15) | 58.5 (30) | 72 (30) | 35 |
|  | *C. labrosus* |  | *efl1α*qF2 | *efl1α*qR1 | “ | “ | “ | “ |
|  | *P. lascaris* |  | *efl1α*qF3 | *efl1α*qR1 | “ | “ | “ | “ |
|  | *A. presbyter* |  | *efl1α*qF3 | *efl1α*qR1 | “ | “ | “ | “ |
